# *Haliotis discus discus* Sialic Acid-Binding Lectin Reduces the Oncolytic Vaccinia Virus Induced Toxicity in a Glioblastoma Mouse Model

**DOI:** 10.3390/md16050141

**Published:** 2018-04-26

**Authors:** Gongchu Li, Shengsheng Mei, Jianhong Cheng, Tao Wu, Jingjing Luo

**Affiliations:** College of Life Sciences, Zhejiang Sci-Tech University, Hangzhou 310018, China; mss1053280369@163.com (S.M.); cjh15067149909@163.com (J.C.); wutao0920@163.com (T.W.)

**Keywords:** HddSBL, oncolytic vaccinia virus, glioblastoma, adverse effects

## Abstract

Although oncolytic viruses provide attractive vehicles for cancer treatment, their adverse effects are largely ignored. In this work, rat C6 glioblastoma cells were subcutaneously xenografted into mice, and a thymidine kinase-deficient oncolytic vaccinia virus (oncoVV) induced severe toxicity in this model. However, oncoVV-HddSBL, in which a gene encoding *Haliotis discus discus* sialic acid-binding lectin (HddSBL) was inserted into oncoVV, significantly prolonged the survival of mice as compared to the control virus. HddSBL reduced the tumor secreted serum rat IL-2 level upregulated by oncoVV, promoted viral replication, as well as inhibited the expression of antiviral factors in C6 glioblastoma cell line. Furthermore, HddSBL downregulated the expression levels of histone H3 and H4, and upregulated histone H3R8 and H4R3 asymmetric dimethylation, confirming the effect of HddSBL on chromatin structure suggested by the transcriptome data. Our results might provide insights into the utilization of HddSBL in counteracting the adverse effects of oncolytic vaccinia virus.

## 1. Introduction

Glioblastoma is the most common primary brain tumor and shows poor prognosis. The 2-year overall survival of glioblastoma patients is approximately 25% after current standard radiation and chemotherapy [1,2]. The failure of effective treatment for glioblastoma is at least partly due to the restricted permeation of drugs across the blood–brain barrier (BBB) [3,4]. Therefore, novel technologies for safer and effective clinical application are essential.

Oncolytic virotherapy is a promising treatment modality that selectively targets cancerous tissues without harming normal tissues [5]. In this strategy, virus vectors are usually designed by deleting genes important for viral replication in normal cells and inserting therapeutic genes. Through specifically infecting cancer cells, viruses amplify in the tumor and infect more cancer cells [6]. Several viruses, including measles virus (MV) [7], myxoma virus (MYXV) [8], adenovirus (Ad) [9,10], herpes simplex virus (HSV) [11,12,13], and vaccinia virus (VV) [14] have been utilized in glioblastoma treatment. Compared with other viral vectors, vaccinia virus offers several advantages, including large packing capacity of exogenous genes, the ability of overcoming BBB for effective tumor treatment, and rapid replication in cytoplasm, instead of integration into the host genome [15]. Oncolytic vaccinia viruses (oncoVV) have been used in clinical trials, including GLV-1h68 [16] and JX-594 [17,18,19,20]. JX-594 has a deletion of the thymidine kinase gene, and expression of human granulocyte-macrophage colony stimulating factor (GM-CSF) and β-galactosidase (β-gal) proteins, and has showed enhanced cytotoxicity in mouse GL261 glioma cells, compared with reovirus or VSVΔM51. However, the adverse effects of oncolytic viruses have been largely ignored.

Lectins, distributed ubiquitously in plants, animals, and fungi, are highly diverse carbohydrate-binding proteins, which selectively recognize and bind distinct sugar-containing receptors on cellular surfaces [21,22]. Regarding their biochemical properties, lectins hold not only potential for cancer diagnosis and prognosis, but also show great potential for application in cancer therapy, through activating apoptotic- or autophagic-related signaling pathways. Previous studies have showed the anticancer potential of various lectins, including galectin [23,24], mistletoe lectin [25], concanavalin A [26], and MytiLec [27,28,29,30]. In our previous work, we have demonstrated the anticancer efficiency of adenovirus-mediated lectin expression, including mannose-binding lectin from *Pinellia pedatisecta* agglutinin (PPA) [31], *Ulva pertusa* lectin 1 [32], *Strongylocentrotus purpuratus* rhamnose binding lectin (SpRBL), *Dicentrarchus labrax* fucose binding lectin (DlFBL) [33], and *Haliotis discus discus* sialic acid-binding lectin (HddSBL), which elicited significant in vitro and in vivo suppressive effects on a variety of tumor cells. HddSBL exogenously expressed from adenovirus vectors has shown significant growth inhibition on hepatocellular carcinoma Hep3B cells, colon carcinoma SW480 cells, and lung cancer cell lines A549 and H1299 [34].

Here, we show that an oncoVV with the deletion of thymidine kinase gene was toxic to mice subcutaneously xenografted with rat C6 glioblastoma cells. Interestingly, the survival of C6 glioblastoma xenograft mice was prolonged by oncoVV harboring HddSBL (oncoVV-HddSBL) as compared to the control oncoVV virus. We further showed that HddSBL downregulated serum rat interleukin-2 (IL-2) levels, inhibited the production of intracellular antiviral factors, promoted viral replication, and influenced histone methylation.

## 2. Results

### 2.1. HddSBL Reduced the Toxicity of OncoVV in a Subcutaneous C6 Glioblastoma Xenograft Mouse Model

**Figure 1:**
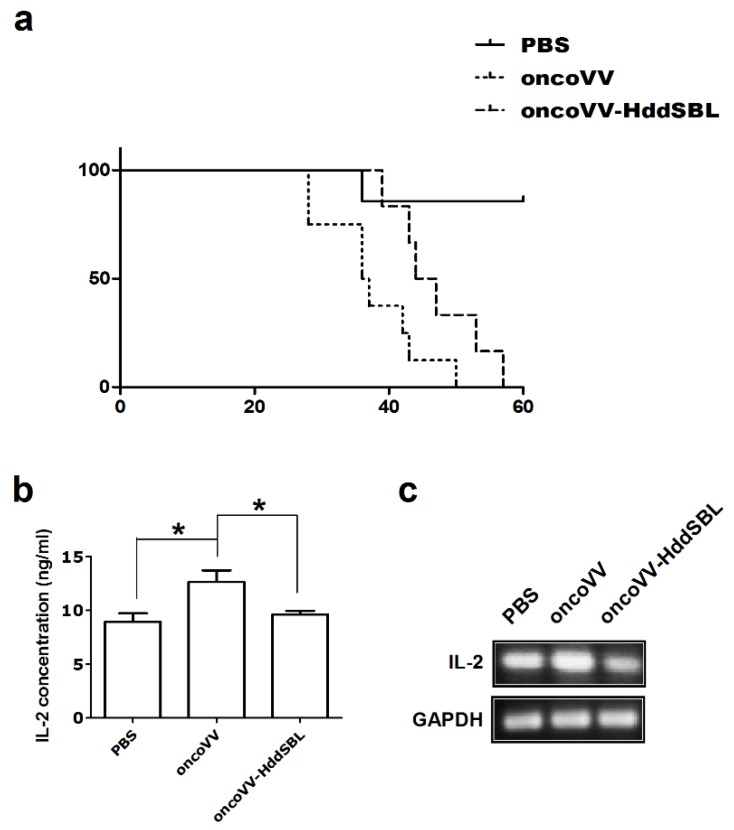
The oncolytic vaccinia virus (oncoVV)-*Haliotis discus discus* sialic acid-binding lectin (HddSBL) reduced toxicity and prolonged survival of mice compared to the oncoVV. (**a**) Kaplan–Meier survival curves of C6 glioblastoma xenograft mouse model. (**b**) ELISA assay of IL-2 secretion. (**c**) Reverse transcriptase-polymerase chain reaction (RT-PCR) analysis of rat IL-2 at mRNA levels.

We first assessed the efficacy of oncoVV and oncoVV-HddSBL in a subcutaneous glioblastoma xenograft model. Rat C6 glioblastoma xenografts were grown in the right flank of athymic BALB/c nude mice. The xenograft model was established by day 10 and the tumor volume reached about 100 mm^3^, followed by intraperitoneal injection of PBS, oncoVV, or oncoVV-HddSBL. As shown in Figure 1a, oncoVV exhibited severe toxicity. However, the survival of the oncoVV-HddSBL group was significantly prolonged as compared to the oncoVV group. Our unpublished data have demonstrated the safety of this control oncoVV virus to several other tumor-bearing mouse models, indicating that the toxicity of oncoVV shown here was induced through acting on C6 tumors. Therefore, we further investigated the effect of oncoVV-HddSBL on C6 xenografts, as well as C6 tumor cells. 

### 2.2. OncoVV-HddSBL Reduced Tumor Secretion of Rat IL-2

**Figure 2:**
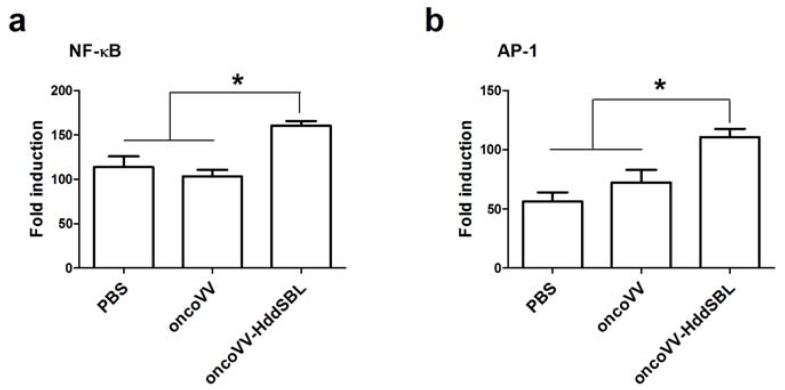
The effects of oncoVV and oncoVV-HddSBL on (**a**) NF-κB and (**b**) AP-1 activation in C6 glioblastoma cells using NF-κB or AP-1 reporter assay. * *p* < 0.05.

We then investigated the potential mechanisms underlying prolonged survival of mice by oncoVV-HddSBL. After 15 days of the first injection of VV, secretion of rat IL-2 in the xenograft tumors was measured by ELISA assay (Figure 1b). Compared to the PBS control, the oncoVV enhanced the secretion of IL-2 (*p* < 0.05), while the oncoVV-HddSBL significantly reduced the secretion of IL-2 compared to the oncoVV group (*p* < 0.05). The transcription levels of rat IL-2 in vitro were investigated by RT-PCR analysis after C6 cells were infected with 5 multiplicity of infection (MOI) of VVs (Figure 1c), which was consistent with ELISA assay results. Furthermore, the activity of inflammation related transcription factors nuclear factor-κB (NF-κB) and activator protein-1 (AP-1) was upregulated in oncoVV-HddSBL-treated C6 cells, as compared to PBS and oncoVV controls (Figure 2). Taken together, our data suggested that the prolonged survival of C6 mice by oncoVV-HddSBL might be due to the significant reduction of IL-2 secretion from tumor cells, whereas the activation of inflammatory transcription factors NF-κB and AP-1 limited this effect.

### 2.3. Virus Replication in Rat C6 Glioblastoma Cells

**Figure 3:**
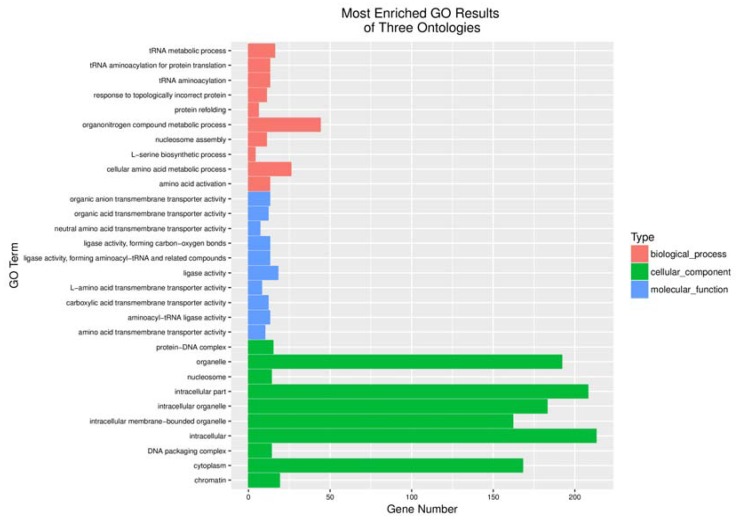
Functional categorization of up-regulated genes based on gene ontology (GO) annotations between oncoVV and oncoVV-HddSBL treatments.

**Figure 4:**
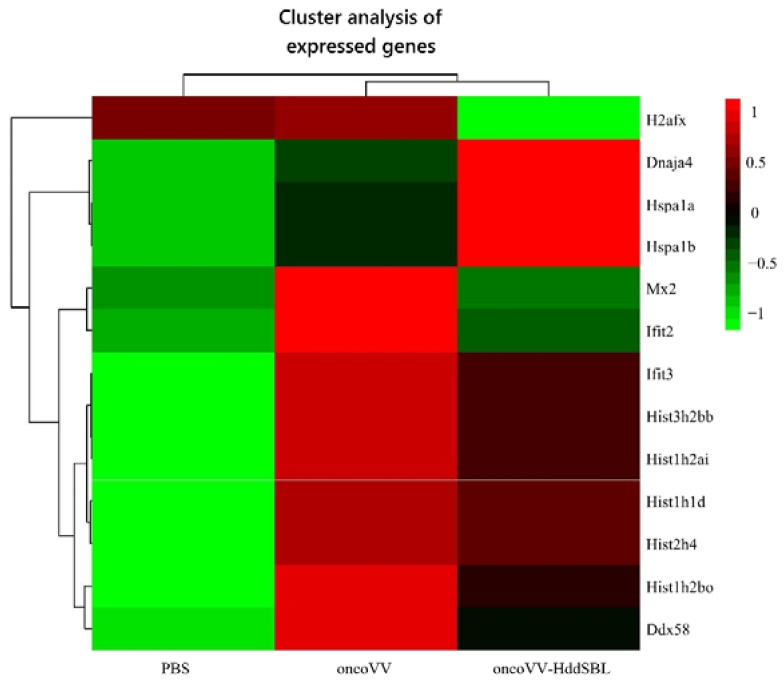
Screening and functional analysis of differentially expressed genes after treatment of C6 glioblastoma cells with PBS, oncoVV (5 MOI), and oncoVV-HddSBL (5 MOI) for 36 h. Scale bar is in log10.

**Figure 5:**
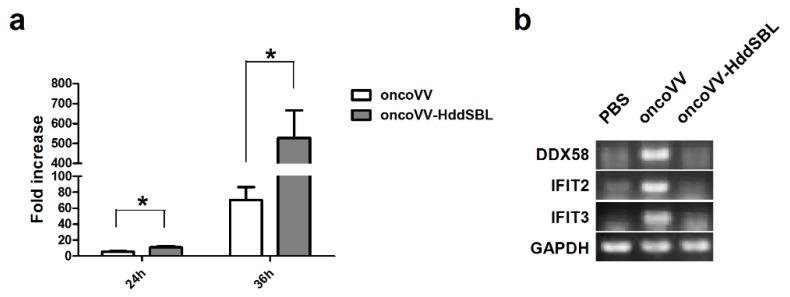
HddSBL promoted viral replication and inhibited antiviral factors in C6 glioblastoma cells. (**a**) Viral replication in C6 glioblastoma cells; (**b**) RT-PCR for mRNA levels of antiviral genes. * *p* < 0.05.

After rat C6 glioblastoma cells were infected with 5 MOI oncoVV or oncoVV-HddSBL for 36 h, total RNA was extracted from cells, differentially expressed genes were screened, and transcriptome sequencing analysis was carried out. The result of gene enrichment analysis is shown in Figure 3. Then, the representing differentially expressed genes were selected and shown in Figure 4, including several factors related to intracellular viral controlling. Therefore, C6 glioblastoma cells were then infected with 5 MOI oncoVV or oncoVV-HddSBL, and virus replication was investigated at 24 h and 36 h. The results showed that the oncoVV-HddSBL was nearly 2-fold higher at 24 h and 7-fold higher at 36 h than that of control oncoVV (Figure 5a). We then investigated the transcription levels of antiviral factors IFIT2 (interferon-induced protein with tetratricopeptide repeats 2), IFIT3, and DDX58 (DEAD-box helicase 58) by RT-PCR. The transcription of IFIT2, IFIT3, and DDX58 was upregulated in oncoVV group, while the oncoVV-HddSBL dramatically decreased their levels as compared to oncoVV treatment (Figure 5b), which was consistent with the transcriptome data shown in Figure 4. 

### 2.4. The Effect of HddSBL on Histone Modification

**Figure 6:**
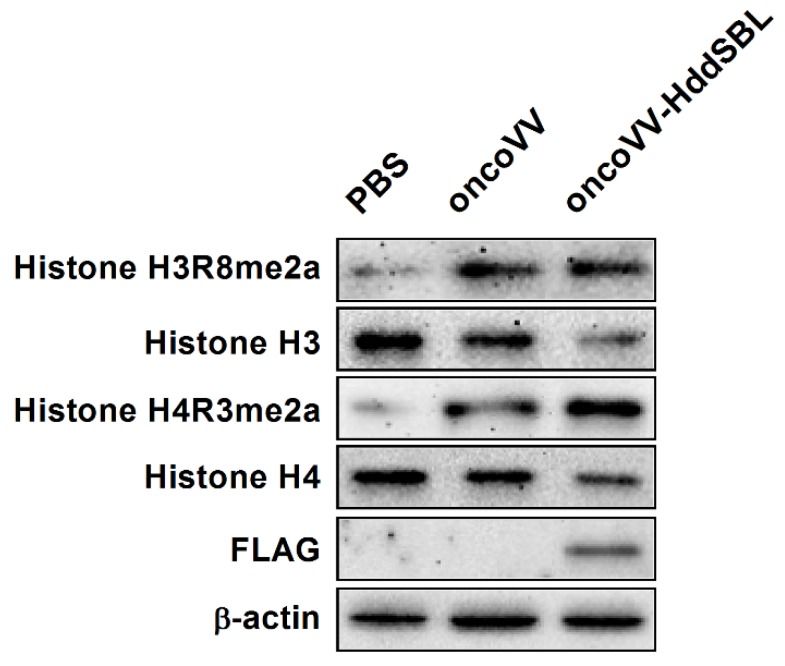
The effect of oncoVV-HddSBL on histone modification. C6 glioblastoma cells were treated with PBS, 5 MOI of oncoVV or oncoVV-HddSBL, and histone H3, H4, H3R8, and H4R3 asymmetric dimethylation levels, as well as the expression of FLAG-tagged HddSBL were analyzed by Western blot.

As shown in our transcriptome data, among the most significant categories, we found terms related to chromatin structure such as “nucleosome”, “protein–DNA complex”, “DNA packaging complex”, and “chromatin”. We then verified the effect of HddSBL on chromatin structure regulation. The oncoVV-HddSBL treatment showed significant downregulation of histone H3 and histone H4, and upregulation of histone H3 Arg8 asymmetric methylation (H3R8me2a), and histone H4 Arg3 asymmetric methylation (H4R3me2a) in C6 glioblastoma cells (Figure 6). Our results indicated that HddSBL influenced histone modification, which was consistent with the result of transcriptome data. Furthermore, the expression of FLAG-tagged HddSBL was also verified by Western blot with an antibody against FLAG.

## 3. Discussion

Oncolytic viruses provide an alternative tool for cancer treatment. The transgenes can be integrated into recombinant vectors to form tumor-selective, multi-mechanistic antitumor agents. Deletion of viral genes that are necessary for replication in normal cells greatly enhances cancer cell-specific replication of oncolytic viruses [35]. Their oncolytic effects can be enhanced through the insertion of foreign antitumor genes [36,37]. Due to the large packing capacity of exogenous genes, VV is particularly attractive as a potential therapeutic agent for the treatment of malignant tumors. However, the adverse effects of VV have been ignored in many studies. In this work, we demonstrated the potential for lectin HddSBL carried by oncoVV for reducing the oncoVV-induced severe toxicity in treatment of glioblastoma. 

The interferons (IFNs) induced protein with tetratricopeptide repeats (IFITs) family participates in diverse processes in response to viral infection [38]. IFIT2 is located in microtubules, and plays an important role in cell proliferation and microtubule dynamics. IFIT3 is located in the cytoplasm and mitochondria, and is also recognized as an antiviral protein. Our study showed that HddSBL inhibits the oncoVV-induced antiviral factors, which favored oncoVV replication in C6 cells. In addition, previous studies have demonstrated that a high dose of IL-2 led to substantial acute toxicity [39,40]. In our study, a decrease in IL-2 secretion was shown to be associated with prolonged survival of oncoVV-HddSBL-treated mice. Thus, our results have suggested that HddSBL affected multiple signaling pathways related to immune responses induced by oncoVV. Therefore, further investigations into the underlying mechanism may help to develop oncoVV-HddSBL into an agent for controlling oncoVV toxicity.

## 4. Materials and Methods

### 4.1. Cell Culture and Production of oncoVV-HddSBL

Rat C6 glioblastoma cells and human embryonic kidney cells HEK293A were obtained from American Type Culture Collection (Rockville, MD, USA) and were cultured in DMEM medium (Gibco, Thermo Fisher Scientific, Waltham, MA, USA) with 10% fetal bovine serum (FBS, Gibco). The gene encoding *Haliotis discus discus* sialic acid-binding lectin (HddSBL, GenBank accession No. EF103404) was integrated into the plasmid pCB with a thymidine kinase (TK) gene deletion to form pCB-HddSBL. The plasmid was cotransfected into HEK293A cells using Effectene Transfection Reagent (Qiagen, Hilden, Germany) with WR vaccinia virus to generate oncoVV-HddSBL through homologous recombination. OncoVV without transgene has been constructed previously as control virus. The viruses were amplified in HEK293A cells and purified by sucrose-gradient ultracentrifugation. 

### 4.2. Subcutaneous C6 Glioblastoma Xenograft Mouse Model

Mice were cared for in accordance with the Guide for the Care and Use of Laboratory Animals. Xenograft tumors (rat C6 glioblastoma) were established by injecting 1 × 10^5^ cells in 100 μL PBS subcutaneously into the right flank of 4–5 weeks old female BALB/c nude mice (Shanghai Slack Animal Laboratory, China), with each group consisting of 7 mice. Treatment started when tumor size reached about 100 mm^3^. OncoVV or oncoVV-HddSBL was injected intraperitoneally at 1 × 10^7^ plaque-forming units (pfu) in 100 μL PBS twice. Control animals received intraperitoneal injections of PBS. The survival of mice was monitored every day. 

### 4.3. ELISA Assay for IL-2 Secretion

The secretion of IL-2 in tumors was determined by ELISA assay using the Rat IL-2 ELISA Kit (Multi Science, CA, USA) according to the manufacturer’s instructions. Briefly, mice serum samples were obtained from tumor veins after 15 days of the first injection of VVs. Serum samples were incubated with anti-Rat IL-2 antibody in ELISA plate for 1.5 h at room temperature. Then, the samples were washed 6 times with washing buffer and incubated with streptavidin-HRP for 0.5 h at room temperature. After washing three times in washing buffer, samples were incubated with substrate solution for 30 min at room temperature. The absorbance of the sample at 450 nm was read on an absorption spectrophotometer after the addition of stop solution.

### 4.4. Screening and Functional Analysis of Differentially Expressed Genes and Analysis of Gene Enrichment

Rat C6 glioblastoma cells were plated at 5 × 10^6^ in 10 cm dishes (*n* = 3). After culture overnight, cells were infected at a multiplicity of infection (MOI) of 5 with oncoVV or oncoVV-HddSBL for 36 h. PBS served as the negative control. Total RNA was extracted from cells using TRIzol reagent (Invitrogen, Waltham, MA, USA). Differentially expressed genes were screened, and the transcriptome sequencing and analysis was carried out by Vazyme Biotech Co., Ltd. (Nanjing, China). 

### 4.5. Virus Replication Assay

To determine the viral replication capacity of VVs in C6 glioblastoma cells, cells were plated on 24-well plates at 1 × 10^5^ cells per well one day before treatment with viruses. Then, cells were infected with oncoVV or oncoVV-HddSBL at a MOI of 2 for 2 h, 24 h, and 36 h. Cells and culture medium were collected and lysed with three cycles of freeze-thawing at the time interval indicated. Then, the supernatants were collected by centrifugation, and viral titers were measured through tissue culture infectious dose (TCID50) assay.

### 4.6. Semi-Quantitative Reverse Transcription Polymerase Chain Reaction (RT-PCR) Analysis

C6 glioblastoma cells were seeded at a density of 8 × 10^4^ cells per well on the 24 well plate. MOI of 5 oncoVV or oncoVV-HddSBL were added, respectively, the next day. Total RNA was extracted from cells using TRIzol reagent (Invitrogen) according to the manufacturer’s instructions. The total RNA was then reverse transcribed into cDNA using reverse transcription kit (TOYOBO). Primer sequences for GAPDH used were 5′-ATGGTGAAGGTCGGTGTGAAC-3′ (sense) and 5′-ATGGGTTTCCCGTTGATGAC-3′ (antisense). The PCR primers for rat IL-2 were 5′-ATGTACAGCATGCAGCTCGC-3′ (sense) and 5′-GATATTTCAATTCTGTGGCC-3′ (antisense). The PCR primers for IFIT3 were 5′-CCATTGCCATGTACCGCCTA-3′ (sense) and 5′-GCATCTTCAACCAACCGCTC-3′ (antisense). The PCR primers for IFIT2 were 5′-ATGCCACTTCACCTGGAACC-3′ (sense) and 5′-CTTCGGCTTCCCCTAAGCAT-3′ (antisense). The PCR primers for DDX58 were 5′-TGCAAGGCGCTCTTTCTGTA-3′ (sense) and 5′-CAAAGCCTTCAAACCTCCGC-3′ (antisense).

### 4.7. Western Blot Analysis

Cells were plated at 1 × 10^6^ in 60 mm dishes. After infected with MOI of 5 oncoVV or oncoVV-HddSBL respectively, cells were harvested in ice-cold cell lysis buffer (Beyotime Institute of Biotechnology, Shanghai, China). The extracts were then subjected to SDS-PAGE and transferred to nitrocellulose membranes. The membranes were subsequently blocked with 5% bovine serum albumin for 2 h at room temperature and incubated at 4 °C overnight with corresponding antibodies. After washing with TBST buffer (0.01 M Tris-buffered saline with 0.1% Tween-20), the membrane was incubated with HRP-conjugated secondary antibodies for 1 h at room temperature. After washing with TBS buffer, membranes were exposed to the Tanon 5500 chemiluminescence image system (Tanon Inc., Shanghai, China). Anti-histone H3, anti-histone H4, anti-flag, and anti-β-actin antibodies were obtained from Cell Signaling Technology Inc. (Danvers, MA, USA). Histone H3 dimethyl Arg8 asymmetric and Histone H4 dimethyl Arg3 asymmetric antibodies were purchased from Active Motif (Carlsbad, CA, USA).

### 4.8. Reporter Assay

To determine the impact of viruses on NF-κB and AP-1 activation, we co-transfected rat C6 glioblastoma cells with the *Renilla* luciferase control plasmid together with reporter plasmids coding for firefly luciferase gene downstream of NF-κB or AP-1 binding sites, followed by treatment of C6 glioblastoma cells with PBS, oncoVV (MOI 5), or oncoVV-HddSBL (MOI 5) for 24 h (*n* = 3). The ratio of firefly to *Renilla* luciferase activity was measured using a dual-luciferase assay system (GeneCopoeia, Inc., Rockville, MD, USA). 

### 4.9. Statistical Analysis

Statistical significance was determined with Student’s *t*-test. *p* < 0.05 was considered significant.

## 5. Conclusions

In this work, a subcutaneous C6 glioblastoma xenograft model was established, and oncoVV-HddSBL exhibited the ability to prolong the survival of tumor-bearing mice as compared to the control virus oncoVV. The tumor secreted serum IL-2 level was downregulated in the oncoVV-HddSBL group compared to the oncoVV group. Furthermore, oncoVV-HddSBL exhibited higher viral replication capability, and intracellular antiviral factors, including DDX58, IFIT2, and IFIT3, induced by oncoVV, were dramatically decreased by HddSBL. HddSBL was also shown to modulate the histone modification and may influence the chromatin structure. Taken together, HddSBL reduced the oncoVV induced toxicity in a C6 glioblastoma mouse model by affecting multiple signaling pathways.
